# Patient-reported experiences and outcomes following hospital care are associated with risk of readmission among adults with chronic health conditions

**DOI:** 10.1371/journal.pone.0276812

**Published:** 2022-11-02

**Authors:** Diane E. Watson, Sadaf Marashi-Pour, Bich Tran, Alison Witchard

**Affiliations:** Bureau of Health Information, Sydney, New South Wales, Australia; Taipei Medical University, TAIWAN

## Abstract

This study quantifies the association between patient reported measures (PRMs) and readmission to inform efforts to improve hospital care. A retrospective, cross-sectional study was conducted with adults who had chronic obstructive pulmonary disease (COPD) or congestive heart failure (CHF) and were admitted for acute care in a public hospital in New South Wales, Australia for any reason (n = 2394 COPD and 2476 CHF patients in 2018–2020). Patient- level survey data were linked with inpatient data for one year prior to risk-adjust outcomes and after discharge to detect all cause unplanned readmission to a public or private hospital. Ninety-day readmission rates for respondents with COPD or CHF were 17% and 19%. Crude rates for adults with COPD were highest among those who reported that hospital care and treatment helped "not at all" (28%), compared to those who responded, "to some extent" (20%) or "definitely" (15%). After accounting for patient characteristics, adults with COPD or CHF who said care and treatment didn’t help at all were at twice the risk of readmission compared to those who responded that care and treatment helped "definitely" (Hazard ratio for COPD 1.97, CI: 1.17–3.32; CHF 2.07, CI 1.25–3.42). Patients who offered the most unfavourable ratings of overall care, understandable explanations, organised care, or preparedness for discharge were at a 1.5 to more than two times higher risk of readmission. Respect and dignity, effective and clear communications, and timely and coordinated care also matter. PRMs are strong predictors of readmission even after accounting for risk related to age and co-morbidities. More moderate ratings were associated with attenuation of risk, and the most positive ratings were associated with the lowest readmission rate. These results suggest that increasing each patient’s positive experiences progressively reduces the risk of adults with chronic conditions returning to acute care.

## Introduction

Adults with chronic health conditions represent a large and disproportionate share of patients served by health systems in nations with advanced economies, and potentially preventable inpatient stays among this population are estimated to be high and costly [1–4]. It’s well established that high hospitalisation and readmission rates for people with chronic conditions, in tandem with evidence of substantial variation in use of hospitals, means that better care might improve this situation [1, 2, 5]. Therefore, to keep people healthy at home and out of hospital, reducing potentially preventable admissions for adults with chronic health conditions is often a priority for health system reform and indicator of health system performance.

In Australia, potentially preventable hospitalisations are estimated to account for 7% of all admissions [^4^]. Each year there are more than 392,000 potentially preventable hospital bed days for chronic obstructive pulmonary disease (COPD) and 412,000 bed days for congestive heart failure (CHF) [^5^]. Given wide variation in age-adjusted hospitalisation rates for these conditions across geographic regions and vulnerable populations, including Aboriginal and Torres Strait Islander people and those living in areas of social disadvantage, reduction in rates has been a national priority for reform for some time [4–6]. In New South Wales (NSW), the most populated state, hospitalisation rates for these conditions are just below national rates. Adults with these conditions consume almost 10% of hospital bed days each year and unplanned re-admission rates are relatively high and persistent (i.e., two in 10 patients) [7, 8].

In Australia and abroad, reform efforts focus on improving primary care and models of clinical and integrated care across the patient journey. Until more recently, there has been relatively less attention in policy, practice, and literature on identifying opportunities to reduce future use of hospital services by enhancing patient experiences in inpatient settings despite evidence that people with chronic conditions, COPD and CHF in particular, visit hospitals frequently and have relative long lengths of stay [^8^]. Indeed, the identification of levers to reduce unplanned readmission is particularly important in Australia as state governments are system managers and responsible for hospital care, but the federal government assumes responsible for primary care.

Therefore, this project assessed the potential impact of improvements in patient reported experiences in hospital on risk of readmission and identified the types of experiences that could be improved to reduce potentially preventable admissions after recent discharge. The focus is on adults who have COPD or CHF, irrespective of the principal diagnoses or reason for admission, due to their high use of hospital care. The null hypothesis was no association.

## Methods

A retrospective cross-sectional study with longitudinal follow-up was conducted by linking NSW Adult Admitted Patient Survey (AAPS) for people who have COPD or CHF with Admitted Patient Data Collection (APDC) which is a census of all admissions to public and private hospitals for a population of 7.5 million and with data from the Registry of Births, Deaths and Marriages. Record linkage was carried out by NSW Health’s Centre for Health Record Linkage (www.CHeReL.org.au). Ethics approval was not required under legislation as patients consented to linkage when completing the survey and the resultant information was used to directly inform the management of health services in NSW.

Between 2018 and 2020, all adults with any diagnostic codes of COPD (40+ years of age) and/or CHF (18+ years) who were admitted to public hospitals for any reason were sent an Adult Admitted Patient Survey and two reminders and responded by mail or online. Hospitals in Australia use International Statistical Classification of Diseases and Related Problems, Tenth Revision, Australian Modification (ICD-10-AM) to classify diagnostic information. In NSW the quality of coded data is monitored, validated and subject to audit (see **S1 and S2 Figs in S1 File** for ICD-10-AM codes). All adults admitted for acute care or rehabilitation from January to March 2018 and 2019 or May to July 2020 were invited to participate (i.e., cross section, census survey) two to three months after discharge, except those admitted for maternal or psychiatric care or same day haemodialysis or those who received hospital in the home, were discharged to nursing homes, or had died (i.e., the target population). Those that received a survey in the prior six months were not included [9–11]. Australia’s international borders were closed in March 2020 and the peak number of cases of COVID in the community reached ~200 per day during data collection in 2020. The work was done as part of the NSW’s Bureau of Health Information’s patient survey program—the largest and most comprehensive program of its type in Australia [12]. The questionnaire includes questions identified as valid, reliable, and easy for patients to understand [13].

To ensure the survey responses related to specific hospitalisations, questionnaires had clear instructions regarding which hospital and month about which ratings of experiences were sought. To identify index admissions to public hospitals and unplanned readmissions to any public or private hospitals in the APDC, survey data were linked with the relevant acute hospitalisations. Around 94% and 93% of the survey responses among the initial COPD and CHF surveyed cohorts, respectively, were linked with a corresponding acute hospitalisation which formed the initial index condition cohorts. Hospitalisation data were compiled for one year prior to risk-adjust the risk of readmission (i.e., Charlson comorbidity score [14] and history of the condition of interest) and hospitalisation and mortality data were compiled after discharge to detect all cause readmission to a public or private hospital. To create a final cohort for analyses, we further excluded adults discharged at own risk, transferred to palliative care or admissions within 90 days following discharge from a prior CHF or COPD index admission. Transfers were considered and multiple acute, contiguous hospitalisations were considered as a single, period of care [15–17]. Around 84% and 86% of the initial COPD and CHF index condition cohorts, respectively, were included in the analyses following these exclusions (**S1 and S2 Figs in S1 File**).

Adults with acute and emergency (un-planned) readmissions within 90 days following discharge from an index admission or, for the purposes of sensitivity analyses, 30 days following discharge from an index admission were identified. Readmissions included patient returns to any public or private hospital following discharge and returns to acute care from non-acute inpatient units or facilities, the later deemed important from a safety and quality perspective but known to represent only 1% of readmissions within 90 days for both cohorts. In cases where more than one readmission occurred within the interested time interval of an index admission, only the first readmission was considered. When there was a non-emergency overnight acute re-hospitalisation within the interested time interval and preceding the first readmission, no readmission was assigned to that index admission as the readmission could be due to the care received during this intervening re-hospitalisation [15, 16].

To identify key aspects of patient experience, a parsimonious set of survey questions were prioritised for analyses based on constructs such as: compassion, respect, kindness and clear communications known to be important to patients, as well as involvement in decision-making and preparedness for discharge known to be important to accreditation [18] and clinical outcomes [19, 20]. To select specific questions we benefited from prior factor analyses of patient surveys used to develop key performance indicators for NSW surveys [21] and literature regarding patient experiences associated with readmissions [22]. Survey questions assessed are included in **Table 1**.

**Table 1:** Patient reported experiences and outcomes assessed for association with readmission to a public or private hospital within 90-days, by clinical condition, 2018–2020.

	COPD	CHF
Overall ratings of care and self-reported outcomes		
Overall, how would you rate the care you received while in hospital?	√	√
Overall, how would you rate the doctors who treated you?	√	NS
Overall, how would you rate the nurses who treated you?	NS	NS
Did the care and treatment received in hospital help you?	√	√
If asked about your hospital experience by friends and family how would you respond?	√	√
Compassion, respect, and kindness		
Did you feel you were treated with respect and dignity while you were in the hospital?	*	√
Effective communication and clear communication		
How much information about your condition or treatment was given to your family, carer or someone close to you?	NS	*
During your stay in hospital, how much information about your condition or treatment was given to you?	√	√
Did the health professionals explain things in a way you could understand?	√	√
If you needed to talk to a doctor, did you get the opportunity to do so?	√	NS
If you needed to talk to a nurse, did you get the opportunity to do so?	NS	NS
Did hospital staff take your family and home situation into account when planning your discharge?	NS	NS
Thinking about when you left hospital, were you given enough information about how to manage your care at home?	√	√
Did hospital staff tell you who to contact if you were worried about your condition or treatment after you left hospital?	NS	NS
Involvement in decision-making		
Were you involved, as much as you wanted to be, in decisions about your care and treatment?	NS	NS
Did you feel involved in decisions about your discharge from hospital?	NS	NS
At the time you were discharged, did you feel that you were well enough to leave the hospital?	√	√
Timely and coordinated care		
How would you rate how well the health professionals worked together?	√	√
How well organised was the care you received in hospital?	√	√
Thinking about when you left hospital, were adequate arrangements made by the hospital for any services you needed?	√	√
Did the hospital provide you with a document summarising the care you received in hospital (e.g. a copy of the letter to your GP or a discharge summary)?	NS	NS

Notes: NS indicates the association was not statistically significant

* indicates although the overall p-value was not statistically significant, but there were categories that were associated with readmission.

To assess the association between key aspects of patient experience and readmissions, univariate and multivariable Cox regression models were used to identify co-variates for each experience question accounting for the clustering of patients within the same hospitals and other patient level risk factors. Covariates reflected sociodemographic and health characteristics derived from survey and prior one-year of administrative data to risk-adjust results [15, 16, 23]. A shared-frailty Cox regression model was used, as it incorporates cluster-specific random effects for time to event data. The follow-up time of the study started from date of discharge to the time of readmission, or the end of the study period based on the availability of the linked administrative data (i.e., Jun 2018 for those surveyed in 2018, and December 2020 for those surveyed during 2019–2020), whichever occurred first. Based on the linked mortality data and given death was one of the exclusion criteria, there was no competing risk of death and therefore no need for using competing risks regression models. Separate models were developed for each of the key survey questions and each condition cohorts. Missing or inapplicable responses were excluded. The prevalence of missing responses ranged from 1% to 14%, only four questions having missing responses of more than 5% and only one of these was associated with readmissions. There was no systematic difference between those with missing and non-missing responses for these four questions in relation to patients’ comorbidities (p>0.090) or readmission risk (p>0.107).

Other demographic and clinical factors included in the development of the regression models were age at discharge (continuous, tested for curvilinearity), sex, education level (Less than Year 12 or equivalent; completed Year 12 or equivalent; trade or technical certificate or diploma; university degree, post-graduate/ higher degree), language spoken at home (English language; others), time of survey, as well as previous history of COPD or CHF, Charlson comorbidity score [14], obesity [24], and smoking [24]—all with a one year look back period.

For each of the key survey questions, a backward modelling approach was used to build the multivariable regression models, with the key experience question included as the variable of interest. Other demographic and clinical variables significant at 20% level in the univariate analysis were considered for inclusion in the multivariable models, and only variables with a two-sided P-value of less than 0.05 in the multivariable models were retained in the final models. The effect of excluding variables from the models on other coefficients was also assessed. Variables excluded from the initial multivariable models (not significant in the univariate analysis at 20% level) were then added one by one and retained in the final multivariable models where P < 0.05 [25].

The proportional-hazards assumption was tested based on Schoenfeld residuals and it was not violated. Sensitivity analyses were performed using random effect logistic regression models, forcing age and sex into the models when not significant, and including missing responses with a percentage of more than 5 as a separate category in the models, which all showed consistent results with the main analyses. All analyses were performed using SAS V.6.3 (SAS Institution Inc., Cary, NC, USA) and STATA V.12 (StataCorp, College Station, TX, USA).

## Results

Between 2018 and 2020, 2394 adults with COPD and 2476 adults with CHF who responded to the survey after discharge were included in these analyses. Response rates and consent to data linkage varied annually where 38% of adults with COPD completed the survey and 81% agreed to linkage in 2020 (44%, 71% in 2019 and 43%, 74% in 2018, respectively). In 2020, 40% of adults with CHF completed the survey and 77% agreed to linkage (41%, 72% in 2019 and 42%, 73% in 2018, respectively) [9–11].

Among the COPD cohort, 402 respondents had a readmission within 90-days (i.e., 17%), and 478 CHF respondents had a readmission within 90-days (19%). While the same people were eligible to receive a survey each year, less than 20 people did. Respondents to the survey who had each of the conditions were assessed individually with the general population who admitted to hospitals, in terms of age, sex and indigenous status which showed a similar pattern of demographic characteristics (i.e., low risk of response bias). And, the aim was to explore associations using risk-adjusted models, so the samples were not weighted.

The socio-demographic and health profiles of respondents are provided in **Table 2**. Among the COPD cohort, patients with a higher Charlson comorbidity score, those admitted to hospital during May to July in 2020, or those with a prior history of COPD were at greater risk of readmission within 90-days. Among the CHF cohort, in addition to Charlson comorbidity score and time of admission, older age was associated with a higher risk of readmission. Based on the recorded ICD-10-AM diagnosis codes during the index admissions and readmissions, no patient was identified as COVID-19 positive during 2020.

**Table 2:** Social, demographic, and clinical characteristics for participants who have COPD or CHF, 2018–2020.

	COPD			CHF		
	**Patients n (%)**	**Readmissions n (%)**	**p-value**	**Patients n (%)**	**Readmissions n (%)**	**p-value**
**Total**	2,394 (100)	402 (100)		2476 (100)	478 (100)	
Age, mean (SD)	74.8 (10)	75.6 (9)	0.051	77.6 (11)	79.0 (10)	0.001
**Age group**						
40–54	79 (3)	8 (2)		95 (4)	11 (2)	
55–74	1101 (46)	188 (47)		800 (32)	138 (29)	
75+	1214 (51)	206 (51)	0.272	1581 (64)	329 (69)	0.017
Sex						
Male	1298 (54)	230 (57)		1411 (57)	260 (54)	
Female	1096 (46)	172 (43)	0.186	1065 (43)	218 (46)	0.202
**Language spoken at home**						
Other	176 (7)	31 (8)		310 (12)	61 (13)	
English	2191 (91)	368 (91)		2125 (86)	408 (85)	
Missing	27 (1)	3 (1)	0.702	41 (2)	9 (2)	0.893
**Education level**						
Less than Y12 or equivalent	1224 (51)	207 (51)		1178 (48)	220 (46)	
Y12 or equivalent	349 (15)	69 (17)		351 (14)	79 (16)	
Certificate	557 (23)	83 (21)		589 (24)	119 (25)	
University	173 (7)	26 (6)		240 (10)	36 (7)	
Missing	91 (4)	17 (4)	0.371	118 (5)	24 (5)	0.211
**Smoking**						
No	1861 (78)	304 (76)		2261 (92)	440 (92)	
Yes	533 (22)	98 (24)	0.264	215 (88)	38 (8)	0.526
**Obesity**						
No	2344 (98)	395 (98)		2392 (97)	462 (97)	
Yes	50 (2)	7 (2)	0.594	84 (3)	16 (3)	0.951
**Comorbidity score**						
0	1266 (53)	174 (43)		812 (33)	135 (28)	
1	515 (21)	102 (25)		588 (24)	104 (22)	
≥2	613 (26)	126 (31)	<0.001	1076 (43)	239 (50)	0.005
**Time of survey**						
Jan-Mar 2018	897 (37)	119 (30)		813 (33)	134 (28)	
Jan-Mar 2019	720 (30)	109 (27)		608 (25)	109 (23)	
May-Jul 2020	777 (32)	174 (43)	<0.001	1055 (43)	235 (49)	0.004
**Previous history of COPD**						
No	1781 (74)	237 (59)		2085 (84)	391 (82)	
Yes	613 (26)	165 (41)	<0.001	391 (16)	87 (18)	0.108

In terms of annual admissions to hospital, the 777 adult COPD respondents in 2020 (i.e., the most recent year) had a total of 2710 acute periods of care (i.e., admissions, excluding transfers). Around one in four admissions were for (i.e., with a principal diagnosis of) COPD (23%) and the remainder for other reasons. Throughout that year, around four in ten had one acute admission (38%), three in ten had two acute admissions (29%) and three in ten had three or more acute admissions (33%). Similar patterns of use of hospital were observed among COPD cohorts in 2018 and 2019. In 2020, 1055 adult CHF respondents had a total of 3062 acute periods of care. Around one in four were for CHF (24%) and the remainder for other reasons. Throughout that year, around four in ten had one acute admission (36%), three in ten had two acute admissions (27%) and four in ten had three or more acute admissions (37%). Similar patterns of use of hospital were observed among CHF cohorts in 2018 and 2019.

In terms of patient reported outcomes, crude readmission rates for adults with COPD who reported that care and treatment received in hospital did not help "at all" were 28%, which was higher than rates among those who reported that it helped "to some extent" (20%) or "definitely" (15%). After accounting for patient characteristics that influence ratings on this question and readmission, adults with COPD who reported that care and treatment did not help "at all" were at twice the risk of readmission within 90-days compared to those who responded "yes, definitely" (Hazard ratio (HR) 1.97, CI: 1.17–3.32). Adults with COPD who reported that care and treatment in hospital helped "to some extent" had a 30% higher risk to be readmitted (HR 1.29; CI 1.02–1.62) compared to those who said it "definitely" helped **(Table 3**). Similar results were evident for adults with CHF (**Table 4**).

**Table 3:** Risk-adjusted statistically significant patient experiences associated with 90-day readmissions for adults with COPD, 2018–2020.

Key survey questions	Patients (n)	Readmission (%)	Hazard ratio	p-value	95% Confidence Interval		Overall p-value
Total	2,394	17					
**Overall rating of care**							
Overall, how would you rate the care you received while in hospital?							0.001
Very good	1,679	16	Ref				
Good	548	16	0.97	0.782	(0.76	1.23)	
Neither good/poor	106	30	1.82	0.002	(1.25	2.64)	
Poor/very poor	29	31	2.25	0.018	(1.15	4.43)	
Overall, how would you rate the doctors who treated you?							0.027
Very good	1,608	16	Ref				
Good	567	18	1.12	0.352	(0.88	1.41)	
Neither good/ poor	107	24	1.45	0.074	(0.96	2.18)	
Poor/very poor	26	31	2.49	0.012	(1.22	5.10)	
Did the care and treatment received in hospital help you?							0.007
Yes, definitely	1807	15	Ref				
Yes, some	492	20	1.29	0.032	(1.02	1.62)	
No, not at all	54	28	1.97	0.011	(1.17	3.32)	
If asked about your hospital experience by friends and family how would you respond?							0.003
Speak highly	1905	16	Ref				
Neither highly nor critical	359	18	1.15	0.313	(0.88	1.50)	
Critical	91	31	1.98	0.001	(1.34	2.93)	
**Compassion, respect, and kindness **							
Did you feel you were treated with respect and dignity while you were in the hospital?							0.140*
Yes, always	2031	16	Ref				
Yes, sometimes	276	22	1.32	0.049	(1.00	1.74)	
No	26	19	1.17	0.725	(0.48	2.86)	
**Effective and clear communication **							
During your stay in hospital, how much information about your condition or treatment was given to you?							0.01
Right amount	1866	16	Ref				
Not enough	378	22	1.42	0.005	(1.11	1.82)	
Too much	18	28	1.62	0.291	(0.66	3.96)	
Did the health professionals explain things in a way you could understand?							0.002
Yes, always	1,709	15	Ref				
Yes, sometimes	543	20	1.27	0.037	(1.01	1.59)	
No	46	33	2.35	0.002	(1.38	3.98)	
If you needed to talk to a doctor, did you get the opportunity to do so?							0.025
Yes, always	1,245	16	Ref				
Yes, sometimes	789	20	1.23	0.052	(1.00	1.53)	
No, no opportunity	92	25	1.66	0.022	(1.08	2.57)	
Thinking about when you left hospital, were you given enough information about how to manage your care at home?							0.001
Yes, completely	1,583	15	Ref				
Yes, to some extent	442	21	1.34	0.019	(1.05	1.71)	
No, not enough	121	26	1.85	0.001	(1.28	2.69)	
Involvement in decision making							
At the time you were discharged, did you feel that you were well enough to leave the hospital?							<0.001
Yes	2,057	15	Ref				
No	255	29	2.02	<0.001	(1.57	2.62)	
Timely and coordinated care							
How would you rate how well the health professionals worked together?							<0.001
Very good	1,388	16	Ref				
Good	760	16	0.90	0.349	(0.72	1.12)	
Neither good nor poor	128	23	1.22	0.322	(0.82	1.81)	
Poor/very poor	36	36	2.89	<0.001	(1.64	5.09)	
How well organised was the care you received in hospital?							0.007
Very well	1,594	15	Ref				
Fairly well	704	19	1.26	0.033	(1.02	1.56)	
Not well	64	27	1.94	0.009	(1.18	3.19)	
Thinking about when you left hospital, were adequate arrangements made by the hospital for any services you needed?							0.025
Yes, completely	1,190	17	Ref				
Yes, to some extent	312	24	1.35	0.028	(1.03	1.77)	
No, not adequate	115	24	1.49	0.051	(1.00	2.22)	

Note: Missing/not applicable responses were excluded. Multivariable models adjusted for Charlson Comorbidity score and history of COPD with a one-year look back period, as well as time of survey. Separate models were developed for each question. Consistent results were observed when forcing age and sex into the models.

* indicates although the overall p-value was not statistically significant, but there were categories that were associated with readmission.

**Table 4:** Risk-adjusted statistically significant patient experiences associated with 90-day readmissions for adults with CHF, 2018–2020.

Key survey questions	Patients (n)	Readmission (%)	Hazard ratio	p-value	95% Confidence Interval		Overall p-value
Total	2,476	19					
Overall rating of care							
Overall, how would you rate the care you received while in hospital?							0.009
Very good	1,723	17	Ref				
Good	604	24	1.38	0.002	(1.13	1.69)	
Neither good/ poor	83	23	1.27	0.315	(0.80	2.02)	
Poor/very poor	37	27	1.63	0.132	(0.86	3.08)	
Did the care and treatment received in hospital help you?							<0.001
Yes, definitely	1919	17	Ref				
Yes, some extent	461	26	1.49	<0.001	(1.21	1.84)	
No, not at all	52	31	2.07	0.005	(1.25	3.42)	
If asked about your hospital experience by friends and family how would you respond?							0.002
Speak highly	1986	18	Ref				
Neither highly nor critical	356	24	1.30	0.030	(1.03	1.65)	
Critical	91	31	1.81	0.003	(1.23	2.67)	
Compassion, respect, and kindness							
Did you feel you were treated with respect and dignity while you were in the hospital?							0.007
Yes, always	2104	18	Ref				
Yes, sometimes	264	27	1.49	0.002	(1.15	1.92)	
No	39	23	1.40	0.322	(0.72	2.72)	
Effective and clear communication							
How much information about your condition or treatment was given to your family, carer or someone close to you?							0.083*
Right amount	1,655	18	Ref				
Not enough	268	23	1.36	0.029	(1.03	1.78)	
Too much	16	25	1.33	0.567	(0.50	3.59)	
During your stay in hospital, how much information about your condition or treatment was given to you?							0.015
Right amount	1938	18	Ref				
Not enough	373	24	1.41	0.004	(1.12	1.78)	
Too much	20	15	0.84	0.770	(0.27	2.63)	
Did the health professionals explain things in a way you could understand?							0.029
Yes, always	1,675	17	Ref				
Yes, sometimes	643	22	1.25	0.028	(1.03	1.53)	
No	57	28	1.58	0.074	(0.96	2.62)	
Thinking about when you left hospital, were you given enough information about how to manage your care at home?							<0.001
Yes, completely	1,616	18	Ref				
Yes, to some extent	467	21	1.17	0.178	(0.93	1.48)	
No, not enough	122	33	1.99	<0.001	(1.43	2.77)	
Involvement in decision making							
At the time you were discharged, did you feel that you were well enough to leave the hospital?							<0.001
Yes	2,187	18	Ref				
No	217	33	2.07	<0.001	(1.61	2.67)	
Timely and coordinated care							
How would you rate how well the health professionals worked together?							0.010
Very good	1,412	18	Ref				
Good	816	20	1.10	0.343	(0.90	1.34)	
Neither good nor poor	115	31	1.83	0.001	(1.29	2.59)	
Poor/very poor	47	19	0.99	0.986	(0.51	1.94)	
How well organised was the care you received in hospital?							0.001
Very well	1,662	17	Ref				
Fairly well	711	23	1.34	0.003	(1.10	1.62)	
Not well	67	30	1.80	0.011	(1.14	2.84)	
Thinking about when you left hospital, were adequate arrangements made by the hospital for any services you needed?							0.016
Yes, completely	1,261	19	Ref				
Yes, to some extent	352	24	1.26	0.067	(0.98	1.62)	
No, not adequate	107	29	1.62	0.012	(1.11	2.36)	

Note: Missing/not applicable responses were excluded. Multivariable models adjusted for age, Charlson Comorbidity score with a one-year look back period and time of survey. Separate models were developed for each question. Consistent results were observed when forcing sex into the models. * indicates although the overall p-value was not statistically significant, but there were categories that were associated with readmission.

In terms of patient reported experiences, overall ratings of care among adults with either of the two chronic conditions were associated with readmission. For example, crude readmission rates for adults with COPD who rated overall care as neither good nor poor, poor, or very poor were twice as high as those who rated overall care as very good or good (i.e., 30% compared to 16%). After accounting for patient characteristics that influence ratings on this question and readmission, the two-fold greater risk in readmission within 90-days was still evident. Adults with COPD who rated their overall hospital care as poor or very poor had more than double the risk of readmission (HR 2.25, CI: 1.15–4.43). Adults with COPD who reported that overall care was neither good nor poor were at almost twice the risk of readmission (HR ratio 1.82; CI 1.25–2.64). No difference was observed in risk of readmission between adults with COPD who reported that overall care was good and those who reported overall care was very good. Similar results were evident when overall care was rated based on propensity to recommend to friends and family (**Tables 3 and 4**).

Respect and dignity were important determinants of risk-adjusted readmissions, as were effective and clear communications. For example, adults with COPD or CHF who reported that they were "sometimes" treated with respect and dignity in hospital had a 30 to 50%, respectively, higher risk to be readmitted within 90-days, compared to those who said they were "always" treated this way (HR for COPD 1.32, CI 1.00–1.74; HR for CHF 1.49, CI 1.15–1.92) (**Tables 3 and 4**). Adults with COPD who reported that health professionals did not explain things in a way they could understand had more than twice the risk of readmission, compared to those who said health professionals "always" explained things in this way (HR 2.35, CI 1.38–3.98). Adults with CHF who reported that they were not given enough information about how to manage their care at home when they left the hospital had twice the risk of readmission, compared to those who said they were given "completely" enough information (HR 1.99, CI 1.43–2.77). Ineffective communications were associated with a 25% to more than two-fold increase in risk of readmission, depending on the measure or clinical cohort (**Tables 3 and 4**).

The degree to which health professionals work together and care is well organised were also determinants of risk-adjusted readmissions. Crude readmission rates tell a similar story as, for example, readmission rates for adults with COPD who reported that care was "not well" organised were higher (27%) than those who reported that care was "fairly well" (19%) or "very well" organised (15%). Adults with COPD who offered poor or very poor ratings of how well health professionals work together had almost three times higher risk to be readmitted (HR 2.89, CI 1.64–5.09), and who reported that care was not well organised had two times higher risk of readmission (HR 1.94, CI 1.18–3.19) than those who offered the most favourable ratings, after accounting for patient characteristics (**Table 3**). Similar results were evident for adults with CHF (**Table 4**).

At the time of discharge, adults with COPD or CHF who did not feel well enough to leave the hospital had a two-fold increase in risk of readmission, compared to those that felt well enough (HR for COPD 2.02, CI 1.57–2.62; HR for CHF 2.07, CI 1.61–2.67). Adults with either condition who reported that the hospital did not make adequate arrangements for any services needed when they left, had a 50–60% greater risk to be readmitted compared to those who reported that arrangements were "completely" adequate after accounting for differences in patient characteristics (**Tables 3 and 4**).

Results of analyses of adults readmitted within 30-days of admission were like those measured at 90-days suggesting that the experiences associated with readmissions are similar for people with these chronic conditions irrespective of when the outcome occurs and, potentially, that patients’ recollection and ratings are relatively stable between 30- and 90-days post-discharge. Due to the smaller number of 30-day readmissions the confidence intervals were larger and, accordingly, some of the associations did not reach statistical significance.

## Discussion

Adults with the chronic conditions mostly offered favourable ratings of inpatient care, though many offered more neutral or unfavourable ratings. Based on the risk-adjusted models, adults with the chronic conditions who reported the most unfavourable ratings of whether hospital care helped had a two times higher risk of readmission compared to those who offered the most favourable ratings. Adults who offered the most unfavourable overall ratings, as well as those who offered unfavourable ratings of the extent to which they were offered understandable explanations, organised care or prepared for discharge had a 1.5 to more than two times higher risk of readmission. Other experiences that matter relate to respect and dignity, effective and clear communications, and timely and coordinated care. Together, these results suggest that high users of hospital services were quite perceptive of the impact of inpatient care on their outcomes. It also concords with systematic reviews regarding the impact of improving patient-centred experiences, organisation and integration of care and transitions of care on readmissions [26–31].

The positive association between patient reported experiences and readmissions is in accordance with a similar study of the general adult inpatient population [^22^] though the magnitude of impact among adults with chronic conditions in this study is higher. Our sensitivity analysis showed this could, in part, be explained by the fact that in this study we were able to compare each of the response categories, separately, with the most favourable response, rather than dichotomizing the responses and comparing the best possible response and the rest of the responses. Also, one hypothesis maybe that adults who use more hospital services, and are at high risk of readmission, maybe more discerning in their judgements of the degree to which care processes impact readmission risk. This proposition might also explain why adults with chronic conditions in this study who reported they did not feel well enough to leave the hospital were at twice the risk of readmission, where a similar study of the general inpatient population did not find an association between patients feeling ready for discharge and readmission or death within 30 days [31].

Most key experience measures selected for assessment in this study were highly associated with readmissions even after accounting for differences between patients that influence ratings and readmission rates, such as age and co-morbidities. One explanation maybe the rigorous process used to select patient reported measures, as the key experience measures selected had already been identified as important in literature, relevant to accreditation and suitable as key performance indicators [18–22].

This study has several strengths. To the best of our knowledge, it is the first to explore associations between admitted patient reported experiences and readmission using linked patient-level data in Australia. Accordingly, the validity of results is not related to ecological fallacy. The use of linked hospitalisation data enabled us to identify unplanned readmissions based on clinical judgement and create an extensive list of potential confounding factors (e.g., age, education, and comorbidities) from survey and administrative data to risk adjust outcomes. Linked longitudinal data and a 1-year lookback period allowed us to follow thousands of patients’ entire journeys and enhance the completeness and comprehensiveness of recorded comorbidities.

At the same time, one limitation of this study is the number of adult respondents. While both clinical cohorts included over 2000 adults, most respondents to the survey offered positive ratings of their experiences with care and the number of respondents who offered the most unfavourable response was relatively small. In some instances, this limited the statistical power to detect differences between the most and least favourable responses (e.g., small sample sizes for patients that report not being treated with respect and dignity). There may be other factors not assessed or available in our data that impact experiences or outcomes. The risk model considered important health and social determinants, so it’s unlikely that other factors would be materially different between patients who report difference experiences or fully explain the large differences between groups in readmission.

The magnitude of impact of patient experiences on outcomes, as well as the array of patient reported experiences associated with readmissions, should be of high interest to policy makers and clinicians for several reasons. It highlights the potential benefits of improvements in patient experiences on reducing potentially preventable admissions among adults with COPD and CHF—cohorts that also have high readmission rates. These cohorts represent a sizable share of potentially preventable hospitalisations according to a commonly accepted performance indicator algorithm [4, 5]. Finally, the results reinforce the face validity of patient experiences and readmissions, at least among adults with chronic conditions, as measures of hospital performance.

Most importantly, patient’s experiences in hospital were strong predictors of readmission even after accounting for characteristics such as age and co-morbidities, and there was a clinically significant graded relationship between patient reported measures and readmission. That is, increasingly the degree to which each patient has positive experiences progressively reduces the risk of adults with chronic conditions returning to acute care for any reason. The most unfavourable experiences were associated with the highest adjusted risk of readmission, and more moderate ratings were associated with attenuation of risk. The most positive ratings were associated with the lowest adjusted risk of readmission. Accordingly, improving patient’s experiences in hospitals can go a long way to keeping people healthy at home and reducing future readmissions in the three months after discharge.

## Supporting information

S1 File (DOCX)
