# Supplementary material for: Patient-reported experiences and outcomes following hospital care are associated with risk of readmission among adults with chronic health conditions
Source: PLoS One. 2022 Nov 2;17(11):e0276812. doi: 10.1371/journal.pone.0276812 (PMC9629632; doi:10.1371/journal.pone.0276812)
Supplement: S1 File — (DOCX) [file pone.0276812.s001.docx]

**Supplementary Figure 1 Chronic Obstructive Pulmonary Disease, cohort exclusions 2018-2020**


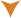

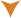


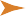

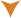

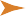

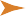

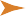

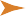


**Initial LBVC surveyed Cohort***

**January – March 2018** Acute/rehab

Principal/secondary diagnosis COPD (J40, J41, J42, J43, J44)

Aged 40+ years

**N =** 1,136 patients

**Linked with an acute hospitalisation**

N=1,088 patients

Discharged at own risk (0.5%)

Index admissions within 90 days of a prior index admission (17.1%)

Transferred to palliative care (0.0%)

In-hospital deaths (0.0%)

Separated from a private hospital (0.1%)

Final COPD index Cohort (2018)

**N =** 897 patients **(**82.4**%)**

**Initial COPD index cohort**

Linked with a COPD acute index period of care

N=1,088 patients


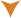

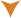


**Initial COPD index cohort**

Linked with a COPD acute index period of care

N=857 patients


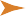

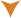

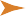

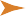

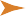

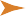


**Initial LBVC surveyed Cohort***

**January – March 2019** Acute/rehab

Principal/secondary diagnosis COPD (J40, J41, J42, J43, J44)

Aged 40+ years

**N =** 957 patients

**Linked with an acute hospitalisation**

N=928 patients

Discharged at own risk (1.0%)

Index admissions within 90 days of a prior index admission (14.1%)

Transferred to palliative care (0.0%)

In-hospital deaths (0.0%)

Separated from a private hospital (1.5%)

Final COPD index Cohort (2019)

**N =** 720 patients **(**84.0**%)**


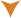

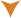


**Initial COPD index cohort**

Linked with a COPD acute index period of care

N=898 patients


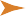

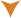

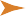

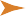

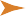

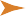


**Initial LBVC surveyed Cohort***

**May – July 2020** Acute/rehab

Principal/secondary diagnosis COPD (J40, J41, J42, J43, J44)

Aged 40+ years

**N =** 939 patients

**Linked with an acute hospitalisation**

N=926 patients

Discharged at own risk (1.0%)

Index admissions within 90 days of a prior index admission (11.2%)

Transferred to palliative care (0.1%)

In-hospital deaths (0.0%)

Separated from a private hospital (1.6%)

Final COPD index Cohort (2020)

**N =** 777 patients **(**86.5**%)**

**Supplementary Figure 2 Congestive Heart Failure, cohort exclusions 2018-2020**


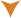

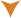


**Initial CHF index cohort**

Linked with a CHF acute index period of care

N=952 patients


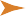

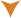

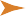

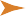

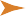

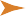


**Initial LBVC surveyed Cohort***

**January – March 2018** Acute/rehab

Principal/secondary diagnosis CHF (I50.0,I50.1,I50.9,I11.0,I13.0,I13.2 )

Aged 18+ years

**N =** 985 patients

**Linked with an acute hospitalisation**

N=952 patients

Discharged at own risk (0.6%)

Index admissions within 90 days of a prior index admission (14.0%)

Transferred to palliative care (0.0%)

In-hospital deaths (0.0%)

Separated from a private hospital (0.1%)

Final CHF index Cohort (2018)

**N =** 813 patients **(**85.4**%)**


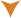

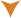


**Initial CHF index cohort**

Linked with a CHF acute index period of care

N=733 patients


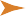

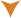

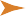

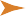

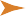

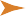


**Initial LBVC surveyed Cohort***

**January – March 2019** Acute/rehab

Principal/secondary diagnosis CHF (I50.0,I50.1,I50.9,I11.0,I13.0,I13.2 )

Aged 18+ years

**N =** 844 patients

**Linked with an acute hospitalisation**

N=817 patients

Discharged at own risk (0.6%)

Index admissions within 90 days of a prior index admission (11.9%)

Transferred to palliative care (0.0%)

In-hospital deaths (0.0%)

Separated from a private hospital (5.5%)

Final CHF index Cohort (2019)

**N =** 608 patients **(**82.9**%)**


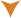

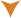


**Initial CHF index cohort**

Linked with a CHF acute index period of care

N=1,199 patients


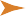

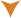

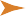

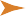

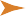

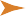


**Initial LBVC surveyed Cohort***

**May – July 2020** Acute/rehab

Principal/secondary diagnosis CHF (I50.0,I50.1,I50.9,I11.0,I13.0,I13.2 )

Aged 18+ years

**N =** 1,278 patients

**Linked with an acute hospitalisation**

N=1,245 patients

Discharged at own risk (0.9%)

Index admissions within 90 days of a prior index admission (9.0%)

Transferred to palliative care (0.0%)

In-hospital deaths (0.0%)

Separated from a private hospital (2.7%)

Final CHF index Cohort (2020)

**N =** 1,055 patients **(**88.0**%)**

Note: Exclusions are not mutually exclusive

*Includes surveyed patients with the condition of interest who consented to the linkage of their survey data to other health datasets
